# Process Mapping to inform implementation of Trauma-Informed Care for youth aged 14–24 with HIV in the Southern United States

**DOI:** 10.21203/rs.3.rs-3234952/v1

**Published:** 2023-08-14

**Authors:** Leslie Lauren Brown, Megan Leigh Wilkins, Latrice Crystal Pichon, Jamie Lynn Stewart, Jessica McDermott Sales, Carolyn Marie Audet, Samantha Veronica Hill, April Christine Pettit

**Affiliations:** Meharry Medical College School of Medicine; St Jude Children’s Research Hospital; The University of Memphis; Meharry Medical College School of Medicine; Emory University School of Public Health; Vanderbilt University Medical Center; The University of Alabama at Birmingham Department of Medicine; Vanderbilt University School of Medicine

**Keywords:** Trauma-informed care, HIV, youth, pediatric, implementation research

## Abstract

**Background:**

Trauma-Informed Care (TIC) is an evidence-based approach for improving health outcomes by providing systematic, trauma- sensitive and -responsive care. Because TIC adoption varies by setting and population, Implementation Science (IS) is particularly well-suited to guide roll-out efforts. Process Mapping (PM) is an IS model for creating shared visual depictions of systems as *they are* to identify rate-limiting steps of intervention adoption, but guidance on *how* to apply PM to guide TIC adoption is lacking. Authors of this study aimed to develop a novel method for conducting TIC-focused PM.

**Methods:**

A real-life TIC implementation study is presented to show how TIC-focused PM was conducted in the case example of a pediatric HIV clinic in a Southern urban area with a high burden of psychological trauma among youth with HIV. A five-phase PM model was applied to evince clinic standards of care, including Preparation, planning and process identification; Data and information gathering; Map generation; Analysis; and Taking it forward. Practices and conditions from four TIC domains were assessed, including Trauma responsive services; Practices of inclusivity, safety, and wellness; Training and sustaining trauma responsiveness; and Cultural responsiveness.

**Results:**

The TIC-focused PM method indicated the case clinic provided limited and non-systematic patient trauma screening, assessment, and interventions; limited efforts to promote professional quality of life and elicit and integrate patient experiences and preferences for care; no ongoing efforts to train and prepare workforce for trauma- sensitive or -responsive care; and no clinic-specific efforts to promote diversity, equity, and inclusion for patients and personnel.

**Conclusion:**

Principles and constructs of resilience-focused TIC were synthesized with a five-phase PM model to generate a baseline depiction of TIC in a pediatric HIV clinic. Results will inform the implementation of TIC in the clinic. Future champions may follow the TIC-focused PM model to guide context-tailored TIC adoption.

## INTRODUCTION

Populations burdened by high rates of psychological trauma require more culturally sensitive health care services to promote optimum wellness. Trauma refers to lasting effects (social, physical, sexual, mental, or spiritual) of adverse events, such as catastrophes (e.g., natural disasters, motor vehicle accidents), personal violations (e.g., sexual assault, racism), or life-threatening situations in which escape is difficult (e.g., HIV diagnosis, child abuse, extreme poverty with housing or food insecurity; American Psychological Association, 2013). According to the Substance Abuse and Mental Health Services Administration’s Treatment Improvement Protocol (TIP) 57, Trauma-Informed Care (TIC) is a multi-level, evidence-based approach for improving health outcomes by preparing care personnel to Realize, Recognize, and Respond to trauma to Resist Re-traumatization (SAMHSA, 2014). Principles guiding TIC include Safety; Trustworthiness and Transparency; Peer Support; Collaboration and Mutuality; Empowerment, Voice, and Choice; and Cultural, Historical, and Gender Issues (SAMHSA, 2014) or Cultural Responsiveness (Brown et al., 2023; Loomis et al., 2018). Resilience-focused TIC is defined as of Organizational Trauma Resilience (OTR) or the extent to which an organization provides the safe, stable, and nurturing environment needed to promote TIC (Brown et al., 2021).

Implementation Science (IS) is well-suited for guiding TIC, but IS has been infrequently applied to TIC adoption (Brown et al., 2021; Han et al. 2021; Piper et al, 2021). Both IS and TIC direct multi-level change efforts as tailored to communities. Community engagement via IS methods offer an apt engine for attuning intervention to context, as is needed to apply the flexible principles of TIC. Further, actionized TIC principles foster democratic and collaborative conditions, with community engagement as a primary vehicle for elevating the voices of many who may have been silenced by structural systems in society. By subsequently implementing a community-adapted intervention, intervention components have been influenced by communities most intimately knowledgeable of a system, which allows implementation activities to be built from leveraged local resources and strengths while tailoring change efforts towards unique gaps and needs.

Process Mapping (PM) is an IS tool for creating shared visual depictions of systems as *they are* to identify rate-limiting steps of intervention adoption (Antonacci et al., 2021; Wagner, 2019). Experientially, conducting PM can also serve to prepare people in systems as well as implementers for intervention adoption as a byproduct of engaging community members to collectively construct shared understandings of systems and discuss areas that could be enhanced by the intervention. A recent systematic review identified a five-phase PM approach, including Preparation, planning, and process identification; Data and information gathering; Process Map Generation; Analysis; and Taking it forward. This five-phase approach includes ten quality metrics related to engaging and educating community members (e.g., patients, personnel, etc.), utilizing standardized symbols, validating the final map, and planning how to build on knowledge gained. However, no guidance could be found on *how* PM may be conducted as part of TIC implementation. Formative research is needed to develop methods by which PM may be systematically conducted to uniquely guide TIC implementation. To fill this knowledge gap, the current study was conducted to create a systematic method for conducting TIC-focused PM.

## METHODS

### Rationale for Current Study

A case example is provided to illustrate how to apply TIC-focused PM. Compared with the general population, persons with HIV (PWH) are exposed to disproportionately more trauma, suffer worse effects, and face unique additional trauma exposures related to diagnosis and serostatus (Sales, Swartzendruber, Phillips, 2016; Friedman et al., 2015), cumulatively contributing to higher rates of Post-Traumatic Stress Disorder (PTSD), disengagement from HIV care, and unsuppressed viremia (Campbell, Raffanti, and Nash, 2019). Extant knowledge suggests these trends are true for PWH of all ages; however, youth with HIV (YWH) experience unique barriers to preventing and addressing trauma and are more likely to endure deleterious neuro-physiological changes, given the trauma burden is occurring during critical years of brain development (Spies et al., 2012).

TIC is as an under-utilized approach with potential to attenuate the syndemic relationship between HIV and trauma. Its implementation has been connected with improvements in patient and provider trauma symptoms and health outcomes (e.g. increases in patient resilience and personnel compassion satisfaction via decreases in burnout; Brown and Vanable, 2018; Sales, Swartzendruber, Phillips, 2016; SAMHSA, 2014) and institutional-level outcomes with adults and youth (Han et al., 2021; Bendall et al., 2020). Despite personnel in HIV care institutions reporting high levels of support for TIC (Piper et al., 2021) and increasing calls to integrate it as part of routine HIV care (End HIV 901, 2021; Piper et al., 2021), there is a paucity of documented efforts to systematically integrate TIC in HIV care settings (Piper et al., 2021). Further, extant trauma interventions insufficiently focus on PWH and primarily focus on patient-level change rather than multi-level system change, with very few studies occurring in the U.S. (Sales et al., 2016) or in pediatric HIV care settings.

### Case Example Setting

Between December 2021 and March 2022, personnel and patient representatives were recruited from a pediatric HIV clinic in an academic medical institution in an urban area in the Southeastern United States. The project was designed by a multidisciplinary team, including external investigators of an epidemiologist, physician scientist, psychologists, two social workers, and an implementation science consultation hub, and an internal, clinic-based psychologist. Recruitment was overseen by the two social workers and clinic-based psychologist. This clinic provides medical HIV care for children, adolescents, and young adults living with HIV, primarily those aged 14–24, with documented high rates of psychological trauma (Radcliff et al., 2007). The institution has capacity for and can provide to patients: transportation, food while on campus, research activities, psychosocial assessments and services (e.g., social work, psychology, psychiatry, chaplain), testing/treatment for sexually transmitted infections (STIs), pharmacy, laboratory, and medical care from advanced practice providers, nurses, physicians, medical assistants/nursing care assistant and patient care representatives. This clinic is funded primarily by charitable donations, U.S. Health Resources and Services Administration Ryan White HIV/AIDS Program as well as research-based funding provided by collaborative groups, foundations and pharmaceutical companies, and fee-for-service payments.

### Approach

An empirically developed five-phase PM method was followed, with attention to ten quality metrics (Antonacci et al., 2021). Metrics include attention to clearly identifying service areas, educating personnel on use of PM, engaging patient representatives in PM, gathering information from varying perspectives to inform the construction of a visual PM, iteratively analyzing and updating the map, utilizing standardized PM symbols, validating the map with key stakeholders, and utilizing the map to implement or test areas of improvement in the system. This process entailed iterative community engagement with personnel and patient representatives of the pediatric clinic to uncover the *who*, *what*, and *how* of clinic workflow. The five phases and action steps taken in the current case example are outlined below, and Table 1 summarizes these methods generally for future researchers and healthcare providers to consider for TIC-focused PM. Though PM data may be collected generally as part of program evaluation, activities conducted for the current case clinic were approved and connected as part of a larger research study by the Meharry Medical College Institutional Review Board (21–07–1105) and University of Memphis (PRO-FY2022–201), with St. Jude ceding to Meharry as IRB of record. The Revised Standards for Quality Improvement Reporting Excellence (SQUIRE) 2.0 checklist was followed as a framework for reporting PM findings as part of healthcare quality improvement (See Additional File 1).

### Phase 1: Preparation, Planning, and Process Identification

Preparation for conducting process mapping began with the overall principal investigator (PI) meeting regularly with clinic site-PI [together multiple-PI (mPI)], which led to receiving verbal and written support for the project from the clinic director. The PI held a hospital-wide Grand Rounds presentation to share broadly with the institution housing the clinic about the significance, principles, and implementation domains of TIC. Plans to engage the community were presented during developmental stages, as part of an established community advisory group, which comprises researchers, practitioners, people living with HIV, and other community members. The multi-PIs held a kickoff meeting with clinic personnel to share information about the intent of the project. The PI met monthly with an NIH-assigned Implementation Science Hub with the University of Alabama at Birmingham Center for AIDS Research to discuss engagement strategies and plans for PM meetings. Finally, demographics and psychosocial characteristics, as well as appointment no-show rates and HIV outcomes of patients were reviewed by mPIs to assess outcomes and potential service areas of greatest need for change.

PM discussion guides were developed from the TIP 57 to guide discussions around four OTR dimensions, including: 1) Trauma Responsive Services, probing for trauma exposure screening and effects assessments and clinical interventions; 2) Practices of Inclusivity, Safety, and Wellness, probing for efforts to promote professional quality of life and elicit patient experiences and preferences for care; 3) Training and sustaining trauma responsiveness, probing for workforce development efforts; and 4) Cultural Responsiveness, probing for efforts to promote diversity, equity, and inclusion at the clinic-level for patients and personnel.

### Phase 2: Data and Information Gathering

PM discussions were conducted first through face-to-face site visits and virtual workgroups. Information was recorded through note taking (e.g., visual observations of physical space, interactions with personnel, interpretation of comments and situations by the study team conducting the site visit) and physical documentation of protocols and instruments employed. Virtual meetings additionally included audio recordings for later review. Meals and snacks were provided during face-to-face meetings to incentivize participation.

Patient representatives were engaged as one group, with all being members of a community advisory group with an existing relationship working with HIV providers in the community to guide care for youth. Members of this group were recruited by study team members via verbal and electronic invitations to participate. Because each member of this group had varying familiarity with the workflow of the HIV clinic, the mPIs utilized an experiential activity to generate feedback about workflow. The group was shown a randomized list of clinic services and activities associated with initial and follow-up patient meetings and asked to prioritize the sequential steps for which they would prefer to receive care and discuss how they would like the care administered. Once preferences were identified, the group was then shown the clinic’s actual current workflow and asked to compare and contrast the two (ideal and actual) systems as a means for eliciting how they might want services to evolve to be more patient-focused or choice-driven.

Introductory personnel meetings focused on fleshing out *who* is responsible for each phase of care and *what* type of care is provided. See **Supplemental Table 1** for a list of topics, questions, and prompts applied during groups. Follow-up groups then focused on gaining a deeper understanding of *how* care is provided, and providers were engaged in small service group discussions so that each discipline/service area could describe their team approach and typical workflow: this included separate meetings for Patient Registration, Clinic Nurses/ Advanced Practice Providers, physicians, social workers/psychosocial services, research assistants, and pharmacists/pharmacy technicians to specifically explore shared understandings of standards of care related to the aforementioned OTR domains.

### Phase 3: Process Map Generation

An initial map was created based on discussions between study mPIs. Standardized PM symbols and structures found in the literature were utilized to construct the map aesthetically (de Bucourt, Busse, Guttler, et al., 2012; McLaughlin, Burke et al., 2014; Pettit et al., 2022).

### Phase 4: Analysis

To analyze findings from PM activities, the map was iteratively refined during and immediately following each work group, to ensure changes were made based on the integration of group member feedback. This was done by reviewing thick notes from discussion meetings and organizing discussion points by the TIP 57 and OTR dimensions. Map drafts were validated or checked for accuracy with a member of each service area as well as the patient representative group.

### Phase 5: Taking it Forward

Results from PM discussions were applied to develop an overall plan for moving TIC implementation forward in the case clinic setting. Findings helped to generate a list of action items, an implementation action plan with timelines, identify individuals who might be responsible for actions, and tailor a one-on-one interview guide to further explore TIC dimensions that presented as too sensitive to be assessed in group discussion formats, including Culture of Trust and Support, Collaboration and Empowerment, and further probe into Cultural Responsiveness. Final PM findings were shared clinic-wide with personnel to generate recommendations for long-term actions (e.g., general policies and procedures to be refined in the clinic) and short-term actions (e.g., best methods for equitably creating a steering committee).

## RESULTS

### Sample

**Figure 1:**
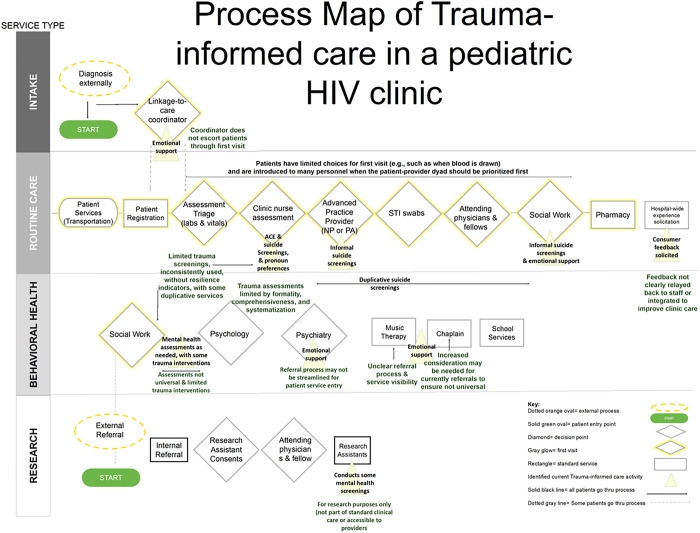
Process Map of Pediatric HIV clinic relative to Trauma-Informed Care

A total of 47 personnel and eight patient representatives participated in PM discussion groups. About a third of participant engagement occurred in-person in December 2021. The remaining group discussions occurred virtually in 2022. The final Process Map is depicted in Fig. 1 and shows who is involved in the system, what type of care is provided, and how it is provided.

#### “Who” is in the pediatric HIV clinic

Clinic personnel were responsible for administering care for a patient census of ~ 250 youth with HIV. Providers included physicians, advanced practice providers (APPs; e.g., nurse practitioners and physician assistants), clinic nurses, support staff (e.g., medical assistants, patient care representatives), mental health practitioners (e.g., social workers, psychologist, psychiatrist, music therapist, child life specialist, educational consultant and chaplain), outreach staff, and pharmacy technicians/pharmacists. Finally, there were research assistants and ancillary personnel working in other campus locations but engaging with patients, including security guards, environmental services, transportation administration and drivers, and patient scheduling.

#### “What” care is provided in the pediatric HIV clinic

APPs reported being primary medical care providers for patients, with physicians conducting twice-weekly rounds and leading clinical staffing discussions in oversight of medical care provided by APPs. Clinic nurses coordinate patient medical care, take histories via health screening assessments and referrals, gather labs, administer medications, and facilitate clinic flow. Medical assistants prepare medical rooms, prepare medical rooms for patients, collect specimens, and take vitals. Patient care representatives register/check-in patients to prepare for visits. Community outreach staff meet patients upon initial diagnosis and link them to clinical care.

All patients were connected with a clinical social worker who provided case management and psychosocial support, including bi-annual psychosocial assessments (e.g., household income, violence in the home, legal proceedings, mental health and substance use history, etc.) and annual Adverse Childhood Experience (ACE) questionnaire screeners (Felitti et al., 2019). Those expressing or showing need for mental health care were referred to the psychologist, who conducted mental health assessments utilizing validated instruments to assess depression and anxiety. Other mental health screeners included a brief depression symptom screener and suicide screener (Posner et al., 2011), administered every six months by clinic nurses. No other routine trauma assessments were conducted with patients. Additionally, individuals needing psychotropic medication management were referred to the psychiatrist who is a consultant holding biweekly afternoon clinics. Research assistants reported regularly conducting validated mental health questionnaires but only for research purposes and results were not integrated into clinical care.

The psychologist was the only mental health provider practicing a trauma-specific patient treatment modality. However, social workers expressed enthusiastic support for TIC implementation and prior interest in/involvement with it. One had certification in trauma intervention and others had training in trauma-sensitive approaches.

When asked about current systems for eliciting/integrating patient and personnel feedback, a hospital-wide satisfaction survey for patients was described but personnel were unsure how the data were utilized or shared. Similarly, hospital-wide employee satisfaction surveys were conducted and shared at larger department-levels, but there were no current practices to elicit clinic personnel satisfaction. Some clinic processes had been amended in response to informal patient verbal feedback (e.g., altering patient screener length), but systematic patient feedback was not routinely gathered or integrated into clinic processes. Personnel and patient representatives suggested the need for clinic administrators to regularly solicit patient feedback (e.g., annual satisfaction surveys) and use it to guide programming. Numerous areas in which patients have authority over their own care were identified, including request for/engagement in psychosocial services, medical care engagement, administration of sexually transmitted infection swabs (e.g., self- or nurse- administered), vaccine uptake, and ART initiation. Regarding workforce development, numerous security guards and nursing administrators were trained every 1.5 years in Crisis Prevention and Intervention to assist when/if a patient becomes escalated behaviorally. When asked about culturally responsive policies and procedures, personnel reported not currently having clinic-specific policies but that the larger hospital system did have some things in place, including basic annual education about legal protections against discrimination, other bias awareness trainings, and opportunities to participate in group discussions and book clubs on cultural topics.

#### “How” care is provided in the pediatric HIV clinic

Patients are seen for routine care every three months to meet with medical providers, get labs drawn, and sometimes receive psychosocial support or engage in clinical research with research staff. Community outreach staff work with new patients to explore potential barriers to care by utilizing Motivational Interviewing (e.g., open-ended questions, affirming responses, and person-centered discussions) (Miller and Rollnick, 2002) to provide education/de-stigmatize HIV. This outreach occurs in medical clinics, patient homes, other public places, or local HIV service organizations.

Annual ACE questionnaires are administered within six months of patients initiating care, often during the second visit. This timeframe was set so sensitive questions would be asked after rapport had been built and psychoeducation provided on childhood adversity (i.e., to reduce unreliable results, as patients were perceived to change answers after establishing relationships). Personnel reported questionnaires were inconsistently conducted—due to limited staff resources and patient and personnel discomfort with the questionnaire items—and no identified structured protocols for how repeated screenings are conducted or systematic methods for sharing information with clinic personnel. Multiple providers, spanning different service categories, reported conducting informal risk assessments of new patients during initial engagement. These topics included verbal questions about current domestic violence risk and/or suicidal or homicidal ideation. Responses were documented in the patient’s medical record. Several personnel, spanning different positions, discussed spending lengthy session times with patients during the first and second appointments to obtain patient history as part of formal and informal psychosocial assessments. Advanced practice providers and physicians identified the need to have improved ease of access to patient responses about trauma exposures, mental health needs, and social determinants of health.

Providers reported engaging in professional development independently, without any clinic-level group trainings being offered specific to TIC and state that leaders would have to elevate this need to ensure clinic-wide training was feasible (i.e., close the clinic during training times or stagger trainings to keep it open with limited staff). Personnel from several positions expressed desire for the social workers’ roles to be expanded beyond primarily case management, into provision of more clinical/therapeutic modalities. A noted benefit to this was that this expansion would be supported as a reimbursable service, which could increase the clinic’s ability to provide greater mental health support.

Initiatives to promote professional quality of life were limited and relied on institution-wide efforts such as tangible treats from a care cart (e.g., teas, coffees, snacks, etc.). Personnel reported appreciating this service but found it alone to be insufficient, occurring infrequently, and not specific to the clinic. Staff burnout and high levels of attrition and turnover were common topics, as was the need for more self-care training and institutional- and clinic-level initiatives to prevent burnout.

The patient representative group generated three recommendations regarding how the clinic might consider integrating patient-centered care, including offering patients a menu of options for the order of services received during first visits (i.e., when blood is drawn that day), limiting the number of providers patients encounter in the first visit, and including a navigator or ambassador to remain with the patient on the first visit to escort them across campus through each appointment during the day.

## DISCUSSION

A systematic method for conducting TIC-focused PM has been presented and includes a synthesis of a five-phase PM model, the TIP 57, and OTR dimensions. Findings from the case clinic are summarized below with comparisons drawn with literature to contextualize findings. Recommendations are provided in-text and summarized in Table 2 for how champions in the case clinic and other settings may conduct TIC-focused PM and build on results. Actions are organized by OTR dimensions—trauma responsive services; practices of inclusivity, safety, and wellness; training and sustaining trauma responsiveness; and cultural responsiveness.

### Trauma Responsive Services

In the case clinic, trauma-related screeners were limited to ACE questionnaires and suicide risk screenings, with additional mental health assessments (e.g., depression and anxiety) provided as *needed* (i.e., not systematically). Strengths of the ACE questionnaire include its parsimony and ability to help identify adversity among youth predictive of future comorbidities (e.g., psychiatric challenges surrounding mental health), so that young patients may be connected with mental health care instrumental in disrupting the formation of comorbidities (Fellitti et al., 2019). Limitations of the ACE questionnaire include its focus on trauma exposures that may not be valid or comprehensive with minority populations in urban settings (Cronholm et al., 2015), an unintentional deficit focus that can occur when not coupled with resilience indicators, and its potential to deflate trauma exposure results because scores are produced by tallying adversities as though each is equally impactful. Hence, ACE questionnaires can be helpful tools when coupled with other instruments.

Practices in the case clinic appear to generally follow federal governing body recommendations; both SAMHSA and Health Resources and Services Administration (HRSA) make recommendations for HIV care to include universal mental health screening (SAMHSA, 2015; HRSA, 2022). Notably, while HRSA produced an executive summary on the importance of addressing trauma as part of HIV care, no concrete requirements or recommendations are included as part of Performance Measures (HRSA, 2015). Consequently, consistent with the case clinic, reports show HIV care providers screen patients for mental health, with a large focus on depression or substance use, but not on trauma exposures or effects. Though a dearth of research explores the prevalence of trauma screeners included in HIV care, a recent study shows 80% of Southern-based community-based HIV organizations provide mental health screenings, with less than half (43%) screening for trauma (Ali et al., 2022).

Considerations for expanding trauma screening practices in the clinic were synthesized from the literature. First, universal trauma Screening, Brief Intervention and Referral to Treatment (SBIRT) approaches are recommended, as conducted by non-clinical staff (e.g., community health workers; SAMHSA, 2015). The SBIRT approach enables trauma identification and referral to treatment while freeing up clinicians to work at the top of their license to administer complex assessments and interventions upon referral. However, this case clinic could reduce duplicative screeners around suicide risk, as excessive discussions around sensitive subjects can be trauma-activating. Second, mental health and trauma assessments should be administered following trauma screeners (SAMHSA, 2016), with trauma assessments requiring at least two instruments to confirm results beyond provisional diagnoses (Connor & Davidson, 2001). Such tools include the PTSD Checklist-5 (Weathers et al., 2013), Impact of Events Scale (Weiss, 200), Clinician Administered PTSD Scale-5 (Weathers, Blake, Schnurr, Kaloupek, Marx, & Keane, 2013), Short Post-Traumatic Stress Disorder Rating (SPRINT; Kim et al., 2008), and International Trauma Questionnaire (Hooper, Stockton, Krupnick, & Green, 2011). However, these assessments should only be conducted with protocols in place to assist patients meeting criteria for PTSD. Third, electronic patient reported outcomes (ePRO) could be utilized to make data available to clinicians (if patients consent to having their data shared electronically). Fourth, resilience-focused assessments should be administered alongside trauma-focused assessments to foster a salutogenic approach (versus pathogenic; Brown et al., 2021). Example resilience assessments include Positive Childhood Experience scale (which is potentially a more robust instrument for predicting mental wellness; Bethell, Jones, Gombojav, Linkenbach, & Sege, 2019), Multilevel Resilience Resource Measure for African American/Black Adults Living with HIV (Dulin et al., 2022), Organizational Trauma Resilience Assessment (for personnel), and Organizational Trauma Resilience – Patient Reported Experience Measure (Brown et al., 2021; Brown et al., 2022). Finally, Plan-Do-Study-Act cycles (Eather, Chiarella, & Donoghue, 2013) could be considered as a means for sharing evaluation findings with stakeholders to inform quality improvement planning.

In the case clinic, the social workers are well poised to provide trauma treatment in conjunction with the psychologist and psychiatrist. Overreliance on social workers for case management services can hinder the expansion of mental health services (SAMHSA, 2021). Numerous interventions have been tested with adults with HIV (Brown et al., 2021; Goldhammer et al., 2021) and could be effective if properly adapted for youth (e.g., prolonged exposure, motivational interviewing, and stress-reducing approaches including meditative, supportive/psycho-educational, cognitive behavioral, and psychopharmacological (Remien, 2019; Watkins, Sprang & Rothbaum, 2018). Consideration for provision of these services should be balanced by potentially lengthy administration times and the need to include peers (living with HIV) and family members.

### Practices of Inclusivity, Safety, and Wellness

In the case clinic, limited efforts were identified as having been intentionally designed to elicit/integrate personnel or patient experiences or promote professional quality of life. According to a recent HIV Service Organization (HSO) study, 55% of HSOs in the South have mechanisms for engaging clients as organizational decision-makers, though it is unclear what / how systematic those mechanisms are, and if results are integrated system-wide (Ali et al., 2022). Services designed without sufficient patient engagement are problematic. Primarily, without these efforts, there is no consistent mechanism for monitoring if patients feel safe in their care institution or if personnel feel safe and valued in their workspace. The need to elevate personnel voices was particularly underscored given that personnel indicated there had been high levels of burnout and turnover. However, high levels of burnout do not appear to unique to the case clinic; recent findings in the Southern U.S. show between 37%-68% of sampled HIV care personnel meet the criteria for burnout or secondary trauma (Ali et al., 2022; Brown et al., 2023). These comparatively high rates are problematic for numerous reasons (Bloom, 2010; Bloom, 2013); burnout (i.e. response to chronic emotional and interpersonal stress) (Friganović et al. 2019, Qiao et al. 2016), often results in job attrition and sub-optimal patient care, including increases in clinical mistakes/errors, depersonalization, and aggressive reactions to patients, which reduces patient satisfaction and engagement in care and contributes to further patient traumatization (Ben-Zur and Michael, 2007; Dall’Ora et al. 2020; Friganović et al. 2019; Jin Jun et al.; Kim et al., 2016; Panagioti et al. 2017; Qiao et al. 2016; Sales et al., 2019). Therefore, efforts to improve professional quality of life and reduce burnout are critical for improving patient outcomes. To address stakeholder decision-making and professional quality of life, TIC implementors can consider convening and working with community-engaged advisory boards or steering committees to promote the co-production of efforts (Graham, Rycroft-Malone, Kothari, McCutcheon, 2022; McLean et al., 2023).

### Training and sustaining trauma responsiveness

Observed clinic findings for trauma training (i.e., inconsistent, not systematic or mandated) are consistent with the literature. A recent study shows only 44% of sampled community-based HIV organizations provide TIC training (frequency or regularity not specified). Importantly, those having received training were ten times more likely to have implemented TIC (Ali et al., 2022), and findings from youth-focused TIC trainings show it has been associated with reductions in provider strain along with positive feedback from both providers and consumers (Suarez et al., as cited in Bendall et al., 2020). Several workforce development approaches are recommended for influencing different levels of system change toward TIC, including the Sanctuary Model (Bloom, 2013) and Safety and Stabilization, the latter developed by/for persons with HIV in the Southern U.S. (Brown et al., 2021).

A significant component of implementing and sustaining a trauma-resilient workforce is conducting evaluation to guide continuous quality improvement. At the patient-level, interventions focus on changing health outcomes and patient perception of care, with a recommended instrument, the Organizational Trauma Resilience – Patient Reported Experience Measure (OTR-PREM), for measuring the extent to which patients find the organization to be safe, stable, and nurturing (Brown et al., 2022). At the provider-level, interventions largely focus on changing personnel knowledge, attitudes, practices, and professional quality of life, with recommended instruments including Attitudes Related to TIC (ARTIC), TICOmeter (practices affecting patients) and Professional Quality of Life (Pro-QOL) scale (burnout, compassion fatigue, and compassion satisfaction) (Hudnall Stamm, 2009). Interventions to improve personnel quality of life might concentrate on concepts from Trauma Stewardship (van Dernoot Lipsky, 2009), in which practitioners learn about trauma exposure response to bring awareness to their need to practice self-care strategies. Some approaches to promoting professional quality of life include provision of on-the-job mental health services for personnel (in addition to off-site employee assistance programs typically offered), reducing clinician bureaucratic duties and concentrating work time on duties that maximize skills at the top of one’s license, and leadership promoting intra-departmental cohesion and fostering an environment in which personnel are encouraged to make autonomous decisions to collaboratively contribute to organizational decision-making (Quenot et al., 2012; Sales et al., 2019). At the organizational level, interventions focus on changing climate and culture, with recommended instruments of the Trauma-informed Climate Scale [TICS] (Hales, Kusmaul, and Nochajski, 2017) and the Organizational Trauma Resilience Assessment (OTRA) (personnel perspective; Brown et al., 2021).

### Cultural Responsiveness

The current case example indicated no specific clinic-based cultural responsiveness efforts were identified, despite racial minorities accounting for 97% of the clinic patient population. These findings are consistent with the literature showing racial trauma is often overlooked during trauma screenings and assessments (Kirkinis, Pieterse, Martin, Agilia, & Brownell, 2021), in interventions (Weiner, 2009; Shankar et al., 2022), and as part of research (Saleem, Anderson, & Williams, 2020; Metzger, Anderson, Are, & Richwood, 2021; Saleem, Anderson, & Williams, 2020). However, anti-Black racism is a documented driver of health inequities (Shankar et al., 2022), with youth of color reporting more racial trauma and having an increased likelihood for experiencing it than non-Black peers. Black youth with HIV, who bear a disproportionate PTSD burden, could benefit from interventions to ameliorate racial trauma sequelae. Some recommendations for promoting cultural responsiveness include integrating systematic efforts to screen/assess/treat racial trauma among patients, assess patient and personnel perceptions of clinic cultural responsiveness, include antiracism as a core component of their value statement and mission, implement antiracism communication practices, and develop policies and procedures that bring greater diversity, equity, and inclusion to the workforce (Bendall et al., 2020). The integration of racial socialization into tailored therapeutic techniques has been shown to promote healing and is positively associated with psychosocial outcomes for Black youth (i.e., increased self-esteem and resilience; Metzger, Anderson, Are, & Richwood, 2021). Recent community-engaged research has helped develop an antiracism practice that may be integrated as a medical provider intervention to promote humanistic communication and disrupt racism as part of clinical encounters (Shankar et al., 2021). The approach includes strength and trust-focused communication techniques such as centering shared humanity and authenticity, addressing racism directly in the moment, and humble inquiry. Future research should explore specific antiracism communication practices that best promote health equity among patients and providers in the HIV clinic setting (Kirkinis et al., 2021).

### Limitations

There are several notable limitations to methods applied during PM discussions. Initial discussions were conducted as mixed groups (i.e., not by department and without attention to power differentials between leaders and personnel) that potentially did not reap the most reliable results; discussions rarely brought attention to challenges or perceived problems, which could be attributed to a perceived fear of leadership reprisal. However, during the second phase of discussions, groups were intentionally conducted by job role to better promote safe dialogue spaces. Future TIC-focused PM should organize groups intentionally to reduce power dynamics that may impede transparent discussions. Because of these group discussion limitations, certain topics requiring more confidential settings—including the OTR dimensions of Culture of Trust and Support and Collaboration and Empowerment—were not explored as part of the current work. Therefore, there may be salient processes that could influence TIC adoption that were not uncovered during PM activities. Hence, process maps should be followed with one-on-one interview, with findings synthesized with PM results. Finally, case study findings only apply to the participating clinic, but the TIC PM methods are hypothesized to be generally applicable for use in other Ryan White-funded clinics in the Southeast U.S.

## CONCLUSION

Findings present a systematic method for conducting TIC-focused PM and provide an illustration of how the method was applied within a clinic serving youth with HIV, who are disproportionately affected by trauma. Personnel and patient representatives of the pediatric HIV clinic were engaged for group discussions, following a five-phase approach, meeting ten quality metrics, to explore the *who*, *what*, and *how* of clinic workflow and validate findings through member checking. Findings enumerate systematic methods for conducting TIC-focused PM in healthcare settings and mark an expansion of the TIC knowledge base. Recommendations for TIC adoption via implementation science have been provided and may be of use for large numbers of clinics. Recommendations focus attention on multiple levels throughout the clinic, underscoring actions that may be taken to enhance patient and personnel experiences, filling a particular gap in knowledge relative to youth-focused trauma-informed HIV care. Overall, findings have the potential to meaningfully inform future TIC implementation.

## Figures and Tables

**Table 1:** Phases, quality criteria, and activities to develop Trauma-informed Care Process Map

Process Mapping phase & quality criteria	Activity

*Preparation, Planning, and Process Identification*	1. Evaluator meets regularly with site champions
1. *Patient service clearly identified.*	2. Kickoff meeting held between clinic personnel and evaluator to share project intent (e.g., Grand Rounds)
2. *Team educated on use of PM*	3. Team consults with Implementation Science (IS) specialists (e.g., NIH-assigned IS Hub)
3. *Patient representative involved*	4. Engage local community via advisory group, comprising researchers, practitioners, persons with lived experience, personnel, and other community members to develop proposal and plan to support work and involve community

*Data and Information Gathering*	1. Review extant literature on process mapping to guide PM activities
1. *Information gathered to inform PM*	2. Review characteristics of clients/ patients to assess outcomes needing improvement
	3. Conduct site visit(s), recording thick descriptions of space and interactions with personnel, including direct care staff and ancillary support staff (e.g., registration, security guards/police officers)
	4. Apply recommendations from TIP 57 to create a list of traumainformed assessment items for site visit (See Supplementary table).
	5. Review written policies and procedures of site
	6. Engage personnel to describe *who* & *what* of services in mixed discipline spaces, with follow-up groups to evince *how* care is provided, conducted by discipline/service area to describe workflow
	a. Groups explore shared understandings of current trauma screenings, assessments, referrals, and services for all care

*Process Map Generation*	1. Create initial map by evaluator & champions
1. *Different perspectives from multiple stakeholder groups gathered*	2. Employ standardized PM symbols

*Analysis* 1. *Process map analyzed*	1. Update map drafts iteratively by reviewing notes from personnel discussions & organizing points by TIP 57 principles & OTR domains for measuring principles in action.
2. *Additional Information gathered and integrated* 3. *Standardized symbols integrated*	2. Review descriptions & decide on summaries for service areas 3. Validate map with a member of each service area & with a patient representative group
4. *Final map validated with key stakeholders*	
*Taking it Forward*	1. Construct white paper/ info-graphic to share with community
1. *Actions based on knowledge gained from PM undertaken, to implement or test areas of improvement*	2. Utilize findings to inform one-on-one qualitative interview guide with personnel & patients to assess perceived barriers & facilitators to TIC adoption in confidential space
	3. Synthesize interview findings with PM results & share in consensus meetings to guide implementation of TIC
	4. Organize TIC Steering Committee to oversee TIC implementation

**Note**: Phases and quality metrics from Antonacci et al. (2021); Trauma-informed care material informed by Substance Abuse and Mental Health Service Administration Treatment Improvement Protocol (TIP) 57 & Organizational Trauma Resilience Assessment (OTRA).

**Table 2:** TIC dimensions, associated activities, & recommendations

OTR dimension	TIC activity	Recommendations
Trauma Responsive Services	Psychological trauma screening	1) integrate universal screening, brief intervention, referral to treatment (SBIRT) for trauma into routine care, with non-clinical staff conducting SBIRT and reducing the number of repeated non-systematized questions on sensitive topics; 2) incorporate mental health and trauma assessments for all positive screens; 3) utilize electronic patient-reported outcomes; 4) integrate resilience assessments; and 5) conduct Plan-Do-Study-Act cycles to improve mental health screening practices in an on-going way.
	Formal trauma assessments	1) trauma screening tools should be followed with gold standard assessment tools following positive screens; 2) multi-level resilience assessments should be integrated; and 3) PTSD assessments should include two-step process to confirm diagnoses.
	Clinical trauma interventions	1) adapt evidence-based approach to setting via implementation science methods; 2) implement intervention(s) to improve patient mental health, access to services, and HIV outcomes, and 3) train more providers to administer different stages of trauma interventions, including paraprofessionals and community health workers; 4) decrease reliance on Licensed Clinical Social Workers for case management, freeing them up to work at the top of license to provide clinical trauma therapy.
Practices of Inclusivity, Safety, and Wellness	Initiatives to promote professional quality of life (Pro-Qol) and culture or trauma resilience	1) implement multi-level interventions to alter institutional climate and culture; 2) utilize instruments as repeated measures (e.g., pre/post/post, etc.) to measure changes in to measure Professional Quality of Life, climate, and culture; and 3) conduct pre-implementation stage research to explore perceived barriers and facilitators to personnel-level TIC interventions; 4) engage advisory board to co-produce TIC implementation efforts.
Training and Sustaining a Trauma Responsive Workforce	TIC training for personnel	1) implement interventions as repeated measures (e.g., pre/post/post, etc) to measure changes in attitudes, practices, etc. to change personnel TIC knowledge, attitudes, and practices; 2) utilize instruments to measure attitudes; and 3) conduct further pre-implementation stage research to explore perceived barriers and facilitators to TIC implementation.
Cultural Responsiveness	Initiatives to promote cultural responsiveness	1) implement interventions to promote humanistic communication among providers and address racism as a form of trauma; and 2) conduct further pre-implementation stage research to explore perceived barriers and facilitators to addressing cultural responsiveness as part of TIC.

## Data Availability

Data sharing is not applicable to this manuscript as data points were not generated through the presented methods.

## Abbreviations

ACE: Adverse Childhood Experiences
APP: Advanced Practice Providers
HRSA: Health Resources and Services Administration (HRSA)
HSO: HIV Service Organization
IS: Implementation Science
mPI’s: Multiple Principal Investigators
OTR: Organizational Trauma Resilience
PM: Process Map
PWH: Persons with HIV
SAMHSA: Substance Abuse and Mental Health Administration
SBIRT: Screening, Brief Intervention, and Referral to Treatment
TIC: Trauma-Informed Care
TIP: Treatment Improvement Protocol
YWH: Youth with HIV
